# Supplementary material for: BATF alleviates ox-LDL-induced HCAEC injury by regulating SIRT1 expression in coronary heart disease
Source: PLoS One. 2024 Dec 16;19(12):e0306514. doi: 10.1371/journal.pone.0306514 (PMC11649077; doi:10.1371/journal.pone.0306514)

Figure 4B_BATF_HCAEC_13.75kDa

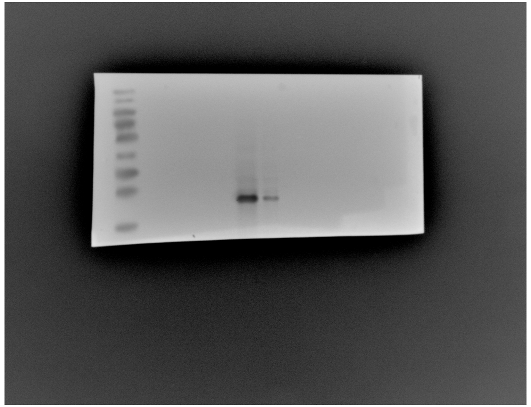

Figure 4B_GAPDH_HCAEC_36kDa

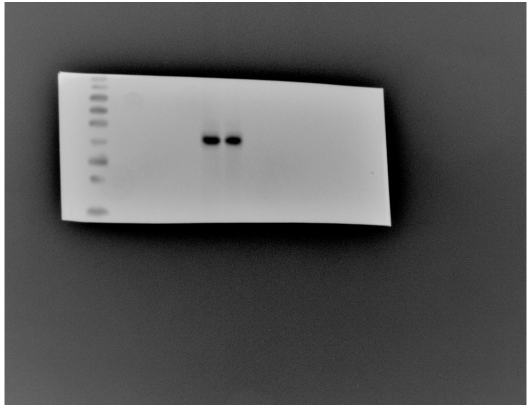

Figure 4F_Bcl-2_HCAEC_26kDa

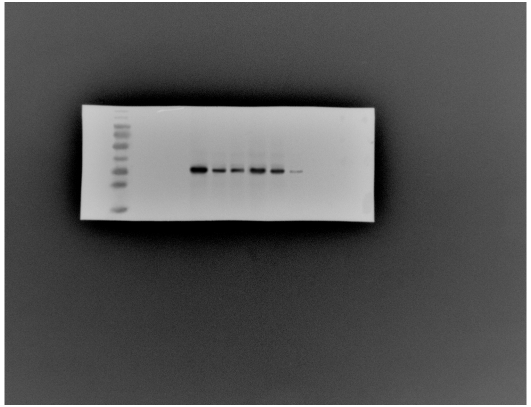

Figure 4F_Bax_HCAEC_18kDa

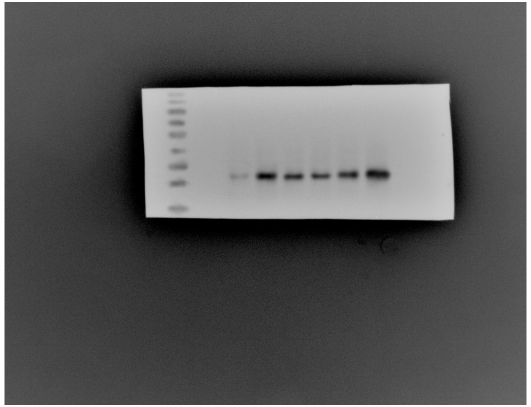

Figure 4F_GAPDH_HCAEC_36kDa

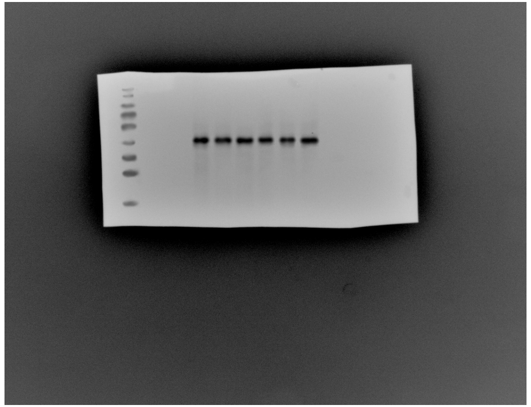

Figure 5B_SIRT1_HCAEC_110kDa

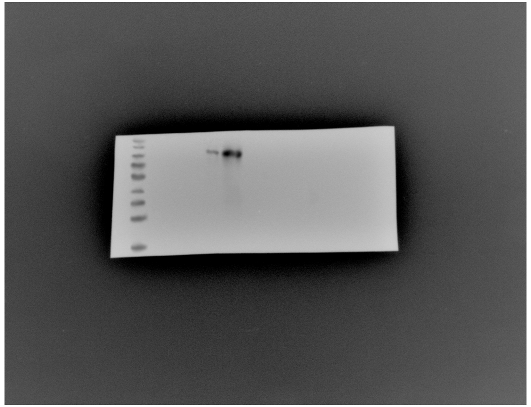

Figure 5B_GAPDH_HCAEC_36kDa

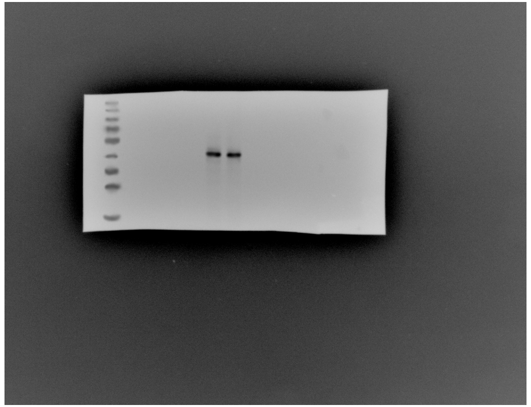

Supplement: S1 Raw images — (PDF) [file pone.0306514.s001.pdf]
