# Supplementary material for: BATF alleviates ox-LDL-induced HCAEC injury by regulating SIRT1 expression in coronary heart disease
Source: PLoS One. 2024 Dec 16;19(12):e0306514. doi: 10.1371/journal.pone.0306514 (PMC11649077; doi:10.1371/journal.pone.0306514)
Supplement: Attachment — Submitted filename: response to reviewers.docx [file pone.0306514.s002.docx]

感谢您披露以下财务信息：“浦东新区科技发展基金民生科研项目（PKJ2023-Y13）、浦东新区卫生健康委员会卫生科技项目（PW2022A-01）、重点学科组” 《浦东新区卫生健康委员会高峰学科建设》（PWZxq2022-11）、《上海市浦东新区卫生健康委员会高峰学科建设》（PWYgf2021-04）》

请说明资助者在本研究中发挥的作用。如果资助者没有发挥作用，请说明：““资助者在研究设计、数据收集和分析、发表决定或手稿准备中没有发挥作用。”

如果此声明不正确，您必须根据需要进行修改。

请将修改后的资助者角色声明包含在您的求职信中；我们将代表您更改在线提交表格
Response: Thank you for your review and valuable suggestions. In response to your proposed modifications regarding the funder acknowledgment, we have made the necessary adjustments and included the revised statement in our article. The modified statement is as follows: "We received key support from funders above for this study. Funding financial support from the Pudong Science and Technology Development Fund and the Pudong Health and Wellness Committee provided the necessary financial security to enable the study to proceed smoothly. These funds were used for study design, data collection and analysis, and preparation of relevant manuscripts. The support of the funders was instrumental in facilitating the progress and success of this study. We sincerely thank them for their generous support and trust." Once again, we appreciate your guidance and attention.

Review Comments to the Author

Please use the space provided to explain your answers to the questions above. You may also include additional comments for the author, including concerns about dual publication, research ethics, or publication ethics. (Please upload your review as an attachment if it exceeds 20,000 characters)

Reviewer #1: I would like to congratulate the authors on their sophisticated work, which will help unraveling some of the secrets of coronary atherosclerosis.
However, I have several comments and points of improvement:
1-Discussion:
The authors did not discuss their results thoroughly or compared their work with the existing literature. Instead, the discussion was a repetition of the introduction, emphasizing on the value of research in this area and the existing gaps of knowledge.
Response: Thank you for your valuable comments. We value your feedback on the discussion section of our paper. We do realize that in the discussion section we did not adequately discuss the results or compare our work with the existing literature. In our revision, we will ensure that our results are analyzed in more depth and compared to the existing literature to better highlight the contributions and innovations of our study. We have revisited the discussion section to ensure that it is different from the introduction section and focuses on the interpretation and analysis of our results, as well as ensuring that it is compared to the relevant literature to better reflect the originality and significance of our study. Once again, thank you for your guidance and suggestions, which we will take seriously and do our best to ensure the quality and integrity of the paper.

2- Results:
the last paragraph (just before the discussion section) is not clear, and there appears to be some mix-up in the presented information (especially in the sentence starting with "Interestingly"). I think you should revise the whole section, and re-write it in a clear and unambiguous way for readers to comprehend.
Response: Thank you for your feedback on my thesis. I will carefully consider your suggestions and revise the results section to improve clarity and accuracy. I have revisited the entire paragraph and ensured that the information is presented clearly and concisely to avoid confusing the reader. Thank you again for your review and valuable comment.

3-Conclusion:
This section is too long, and starts with an opening general statement. I believe it should be much shorter and more concise, and delver the most important finding of this research work.
Response: Thank you for your feedback and suggestions. I fully understand your concerns about this section being too long and starting off too general. I have revisited this section and made it more concise to ensure that it more directly conveys the most important findings of this study. I have eliminated the lengthy content and focused on presenting the core ideas to ensure that readers can clearly understand our findings. Once again, thank you for your valuable comment.

Reviewer #2: The reviewer should modify the title and abstract to elucidate the abbreviations mentioned. The reviewer couldn't understand the meaning of abbreviations presented especially HCAEC.
Response: Thank you very much for your valuable comments and review. I fully understand your concern about the identification of abbreviations, especially for the meaning of HCAEC. I will revise the title and abstract of the paper to explain more clearly all the abbreviations used, including HCAEC. During the revision process, I will make sure that the title and abstract accurately reflect the abbreviations used so that readers can more easily understand what our study is about. Thank you again for your suggestions and patient guidance.
